# HIV Testing Autonomy: The Importance of Relationship Factors in HIV Testing to People in Lusaka and Chongwe, Zambia

**DOI:** 10.1007/s11673-022-10169-9

**Published:** 2022-02-24

**Authors:** Kasoka Kasoka, Matthew Weait

**Affiliations:** 1 HIV Programmes and Advocacy, International AIDS Society, Avenue de France 23, 1202 Genève, Switzerland; 2 University of Hertfordshire, Hatfield, Hertfordshire, AL10 9AB UK

**Keywords:** Autonomy, Common good, HIV response, Human dignity, Informed consent, Dominant traditional African thought and practice

## Abstract

In recent times, informed consent has been adopted worldwide as a cornerstone to ensure autonomy during HIV testing. However, there are still ongoing debates on whether the edifice on which informed consent requirements are grounded, that is, personal autonomy, is philosophically, morally, and practically sound, especially in countries where HIV is an epidemic and/or may have a different ontological perspective or lived reality. This study explores the views of participants from Zambia. In-depth and focus group discussions were conducted at various locations in Lusaka and Chongwe, Zambia. Participants came from various demographics, including people living with HIV (PLHIV), healthcare professionals and workers, policymakers, pregnant women, churchgoers, teachers, rural-based persons, and police officers. Data were manually analysed by conducting inductive and deductive thematic analyses. Results show that participants were not in favour of HIV policies that promote personal autonomy at the expense of pursuit of the common good. Participants viewed interdependence, not autonomy, as an essential characteristic of being human. The participants’ views have a realistic potential to provide a contextual and appropriate ethical, respectful, and realistic foundation for HIV testing policies.

## Introduction

Although much has been accomplished over the last four decades, a great deal still needs to be done to reverse and end the HIV epidemic (Fauci and Lane 2020). For instance, notwithstanding the scientific progress made in HIV prevention and treatment, on September 21, 2020, the Joint United Nations Programme on HIV/AIDS (UNAIDS) reported that the world was unlikely to reach its 2020 90-90-90 targets (UNAIDS 2020a).

These targets aimed to have 90 per cent of people know their HIV status, 90 per cent of those who know their status to receive sustained antiretroviral therapy, and 90 per cent of those on antiretroviral therapy to have viral suppression. Achieving an AIDS-free generation is “more than a historic obligation to the 39 million people who have died of the disease”; it also provides an opportunity to realize a healthier, just, and equitable global society for future generations (UNAIDS 2014b, ¶1). However, it is impossible to realize an HIV-free generation without encouraging HIV testing uptake and bringing treatment to all those who need it (Fire in the Blood 2013; UNAIDS 2014b; Bajunirwe et al. 2018).

The 2021 United Nations Political Declaration on HIV and AIDS reported that the 90-90-90 targets have not been achieved, despite the knowledge and tools the global community now have to prevent new HIV infections as well as AIDS-related deaths (United Nations General Assembly 2021). The global AIDS epidemic thus remains “a global emergency and a paramount health, development, human rights and social challenge” (United Nations General Assembly 2021, 5).

According to UNAIDS, in 2020, 84 per cent of people living with HIV (PLHIV) on average (globally) knew their HIV status, 87 per cent of those were on treatment, and 90 per cent of those on treatment were virally suppressed (UNAIDS 2021a). Overall, approximately 680,000 (estimated range: 480,000-1.0 million) people died of AIDS-related illnesses and 1.5 million (1.0-2.0 million) people became newly infected with HIV in 2020, with new HIV infections increasing in at least thirty-three countries since 2016 (UNAIDS 2021a; United Nations General Assembly 2021).

## HIV and Zambia

HIV prevalence in Zambia has stabilized at high levels (approximately 11.1 per cent of people aged 15–49 years are living with HIV) (UNAIDS 2020c). This makes Zambia one of the countries with the highest HIV burden (Heri et al. 2021). In 2019, 48,000 adults and 5,400 children were newly infected with HIV (compared with 2010 when 47,000 adults and 8,800 children were newly infected), while in 2018, 17,000 people died of AIDS-related illnesses (Avert 2020). As of 2019, 87 per cent of people living with HIV knew their HIV status, 89 per cent were on antiretroviral therapy, and 75 per cent were virally suppressed (all ages) (Avert 2020). These figures indicate that even after eight years of scientific advances, HIV incidence, prevalence, morbidity, and mortality continue to be a major problem in Zambia.

Hence, in August 2017, Zambia’s then president, Mr Edgar Lungu, pronounced: “HIV testing will now be among other tests to be done on patients to ascertain their state of health in order to provide timely and appropriate remedies,” and pledged to aid the country in its efforts to eliminate AIDS by 2030 (Siame 2017, ¶1). The president argued that the policy was justified given that “the HIV/AIDS scourge is one of the biggest threats to the country’s development,” and added that protecting the lives of those affected by HIV “overrides the human rights argument” regarding the need for consent (Siame 2017, ¶3,8). Zambia’s then health minister, Dr Chitalu Chilufya, challenged those opposed to compulsory HIV testing to consider the need to protect the common good—for example, protecting children yet to be born from preventable HIV infections (*The Mast Online*^2017^).

It is important to note at this stage that while compulsory HIV testing may be ethically justifiable in certain circumstances, “particularly when coupled with guaranteed access to treatment and care” (Armstrong 2008, 1), forcing people in any given country to take an HIV test violates human dignity and impedes a successful HIV response (UNAIDS 2021b). Mandatory HIV testing is counterproductive because it prevents and deters people from seeking medical services and learning about their HIV status—especially vulnerable and key populations who already feel stigmatized and marginalized (Avert 2017).

The legality of the reported public policy declaration has not yet been challenged before Zambia’s courts of law. However, in earlier court judgements, for example, *Akashambatwa Mbikusita-Lewanika v Fredrick Chiluba* (1998) and *Kingaipe and Chookole v Attorney-General* (2010), it was held that human persons have a right to consent. Such rights are protected under the Zambian constitution (Republic of Zambia 1996).

## Our Contribution

Our arguments in this paper focus on the importance of continuing to scale up HIV testing to ensure an effective HIV response that will meet the first 90 target (90 per cent of PLHIV know their HIV status) and, looking ahead, the first 95 target (the UNAIDS Fast-Track Strategy to have 95 per cent of PLHIV know their HIV status by 2030) (UNAIDS 2014a). For Zambia to achieve the 95-95-95 targets, it must continue scaling HIV testing uptake.

There are several reasons as to why we have concentrated on the first 90 and 95. HIV testing is critical to the HIV response because individuals can only start treatment if they know their HIV status. In other words, an HIV test result “opens the door to accessing the range of HIV options available depending on a person’s status to keep themselves and their loved ones HIV-free” and forms “the gateway to treatment and effective treatment (UNAIDS n.d..” Moreover, by deciding to know one’s HIV status, “people are empowered to make choices about their right to health” (UNAIDS n.d., 1). Thus, with Zambia still reporting fifty thousand new HIV infections per year (Byanyima 2021), it is imperative to scale up HIV testing to diagnose new infections in the country.

This is particularly imperative in the light of the advent of effective antiretroviral therapy (ART) and its increasing availability at no financial cost in Zambia (Amanzi, Michelo, and Chongwe 2016). HIV infection, which was once a death sentence, is now a serious condition that can be managed with effective treatment. A person living with HIV who is on such treatment and virally suppressed will live longer and is incapable of transmitting HIV to another person.

The undetectable = untransmittable (U=U) doctrine has its foundation in scientific evidence which indicates that PLHIV who consistently maintain undetectable viral loads for a period of at least six months with ART cannot transmit HIV to others (Patel et al. 2020). ART can make the viral load so low that it cannot even be detected by an HIV test—this is what is called an undetectable viral load (Centers for Disease Control and Prevention [CDC] 2021). This means that ART can now be effectively employed as both a treatment and prevention measure. HIV testing and knowing one’s status has therefore become ever more critical in the global HIV response.

Because of this, more innovative HIV testing approaches may be needed to scale up HIV testing in Zambia. To encourage uptake, it may also be necessary to review current HIV testing policies. We therefore invite the reader to consider whether a paradigm shift in HIV testing policies from those exclusively premised on personal autonomy is the way forward in the HIV response, particularly in the sub-Saharan context.

Numerous sub-Saharan countries have adopted HIV-specific laws and policies which have addressed, inter alia, HIV testing. For instance, twenty-six sub-Saharan countries have implemented HIV-specific laws and policies that forbid compulsory HIV testing, including for pregnant women, thereby establishing informed consent as a condition for such testing (Kongnyuy 2009; Eba 2015). This requirement also applies to laws and policies on HIV testing in Zambia (*Kingaipe and Chookole v Attorney-General*^2010^; Kasoka 2018). Moreover, to respect the key principles of informed consent, confidentiality, and other values, Zambia has adopted the World Health Organization’s (WHO) consolidated guidelines on HIV testing (UNAIDS 2017). Consequently, Zambia practices both provider-initiated testing and counselling (PITC) as well as client-initiated counselling and testing (Kasoka 2018), all of which require respect for informed consent.

Although debate is ongoing as to whether informed consent requirements can actually be achieved in practice (Manson and O’Neill 2007), we believe that consent requirements play a crucial role in the response to the HIV epidemic. These include protecting individuals against inhumane and degrading treatment and privacy violations and the protection and promotion of everyone’s right to health and public health (Mann 1996; Kasoka 2018).

Nonetheless, given that the issue of HIV is a human rights concern that can, at best, be legitimately addressed by embracing a comprehensive human rights approach (Gumedze 2004), the question arises as to whether it is ethical to protect individual autonomy with respect to HIV testing without due regard for the rights of others in a given community. To put it another way, is the protection of individual autonomy a justified premise for informed consent requirements? Or is human autonomy so valuable that consent should be premised on this?

## Informed Consent and Autonomy

Several accounts of respect for informed consent in medical ethics claim that informed consent requirements are valuable because they support personal autonomy (O’Neill 2003; Naidoo and Vernillo 2012). As Schuck (1994, 924) notes, “the most fundamental normative argument in favour of requiring health care providers to obtain patients’ informed consent to medical treatments proceeds from the principle of autonomy—the notion that each mature individual has a right to make the basic choices that affect her life prospects.” Faden and Beauchamp (1986) define informed consent as a doctrine premised on the cherished societal value of autonomy that protects self-rule in medical decision-making. Mills (2002, 60) also submits that the argument for consent as an indispensable precursor to treatment is grounded in the concept of patient autonomy, “which in turn is based upon the rights of individual self-determination and of bodily integrity.”

In a landmark high court judgement in Zambia (*Kingaipe and Chookole v Attorney-General*^2010^), Judge Muyovwe ruled that testing individuals for HIV without their informed consent is unlawful. This case established a critical legal precedent because for the first time the court added “its voice to the chorus of recent *obiter dicta* from several jurisdictions in the African region which declared that HIV testing without consent is a violation of human rights as set out in international human rights treaties and other normative instruments” (Malila 2012, 579. In passing her judgement, Judge Muyovwe, among other similar cases, invoked and celebrated the case of *Diau v Botswana Building Society (BBS)*^2003^ (2) BLR 409 (BwlC)*,* where the court held that: “informed consent is premised on the view that the person to be tested is the master of his own life and body” (*Kingaipe and Chookole v Attorney-General*^2010^*, J44*). Thus, “the purpose of informed consent is to honour a person’s right to self-determination and freedom of choice” (*Kingaipe and Chookole v Attorney-General*^2010^*, J44*.

Autonomy is one of the four core principles of modern bioethics, the others being beneficence, non-maleficence, and justice (Lawrence 2007). Respect for autonomy obliges healthcare providers to uphold the choices of patients who have decision-making capacity (Jahn 2011). Beneficence encompasses the following moral obligations: protect and defend the rights of others, prevent harm from occurring to others, remove conditions that will cause harm, and rescue persons in danger (Jahn 2011). Non-maleficence imposes an obligation to do no harm on others. Thus, both beneficence and non-maleficence encompass a number of rules including not to inflict harm or evil, to remove or prevent conditions that will cause harm or evil, and to do or promote good (Naidoo and Vernillo 2012). Finally, the principle of justice obliges health providers to “equitably distribute benefits, risks, costs and resources” (Jahn 2011, 225).

We suggest that the doctrines of beneficence, non-maleficence, and justice impose upon medical health providers a moral duty of care that extends beyond individual patients to society as a whole, resulting in the greatest benefit for all and distributive justice (Faden and Beauchamp 1986; Kinsinger 2009). This would involve implementing HIV testing for the benefit of both the individual and the greater good. This is not necessarily the case with the individual autonomy imperative as respect for individual autonomy begins and ends with an individual. This obliges healthcare providers to respect individuals as responsible for their own person and for their medical therapy, and able to shape their own lives according to their desires and goals (Entwistle, Carter, and McCaffery 2010).

The paramount need for autonomy is a common theme within legal, political, and moral discourse. Thus, “the giving of ‘informed consent’ by a patient has become the surrogate measure of whether medical interventions are ethically” or even legally acceptable (Milligan and Jones 2016, 21). Respect for informed consent requirements is deemed critical if we are to uphold the fundamental value of individual autonomy (Manson and O’Neill 2007), including with respect to HIV testing (Kudzala and Molyneux 2010; Kasoka 2020).

A major premise of autonomy is that individuals are by nature and/or nurture self-governing, self-determining, self-directing, and self-sufficient (Kant 1785; Endleman 1967; Dworkin 1988; Benson 1991; Mele 1995; Oshana 1998; Campbell 2002; O’Neill 2003; Taylor 2018; Christman 2009; Jackson 2013; Mill 2015; Kasoka 2018, 2020). In this paper, autonomy refers to a person’s capacity or ability to self-govern, which includes their moral independence (Oshana 1998; Gaylin and Jennings 2003; Killmister 2013; Mill 2015; Kasoka 2020).

However, is a human person really a “unified, happiness seeking, unbrokenly persisting, ontologically distinct conscious subject who is the owner of experiences, the thinker of thoughts, and the agent of actions” (Zahavi 2014, 42)? And/or is a human a self-moral agent whose autonomy we must protect, even in the context of HIV testing ethics (Kasoka 2020)? Even if it could be demonstrated that an individual is normatively, narratively, and/or morally autonomous, should individual agency take primacy over the collective or common good and due respect of rights of others, particularly in the HIV response in sub-Saharan Africa which disproportionately bears about two-thirds of the global HIV burden (UNAIDS 2020b; 2021a)?

Although extensive textual analyses of the issue of autonomy have been conducted in sub-Saharan Africa and beyond, no empirical study has examined the appropriateness of the value of individual autonomy, especially in the local context of HIV testing in Zambia, and rarely in other sub-Saharan African countries. In this manuscript we seek to answer two questions:

1. What does individual autonomy in HIV testing, and healthcare practice in general, mean to people in Lusaka and Chongwe?
2. What are the implications of participants’ views on promoting the primacy of individual autonomy in medical decision-making in Zambia, and sub-Saharan Africa as a whole?

This study adds to the debate surrounding individual autonomy, including Geoffrey Silavwe’s (1995) article in which he argued that promoting the principle of individual autonomy is inappropriate in the Zambian setting. He asserted that Zambia should not be required to embrace universalized ethics that emanate from and correspond with Western cultures and realities.

## Methods

### Study Design

This exploratory study employed qualitative approaches to investigate the views and lived experiences of participants (Mack et al. 2005; Hancock, Ockleford, and Windridge 2009). Research data were collected from in-depth interviews—a process that facilitated access to more detailed data from individual participants (Kaplowitz 2000; Mack et al. 2005; Boyce and Neale 2006)—and focus group interviews, an approach that afforded the latitude to acquire a complete picture of prevailing social and cultural norms (Elliot and Associates 2005; Mack et al. 2005; Moriarty 2011).

#### In-depth interviews

A total of seventy-three in-depth interviews were conducted. These afforded participants an opportunity to express their views in a way ordinary life rarely affords. In comparison with group interviews, we envisaged and found that in-depth interviews were well suited to acquiring data that would otherwise not be shared in a group setting because it is socially sensitive, highly personal, or both (Mack et al. 2005). The participants came from a range of different backgrounds, including medical professionals and workers, policymakers, service-users, teachers, police officers, churchgoers, pregnant women, PLHIV, people who live in a city, and those in rural areas (see table 1).

**Table 1:** Participant demographics

Variable name	Group	Number of participants
Targeted groups	Pregnant females Teachers PLHIV HIV counsellors Midwives/nurses Medical doctors Policymakers Other*	8 11 8 10 16 6 6 38
Age	18-49 50-73	81 22
Education level	Primary education Secondary education Post-secondary education	11 25 67
Location of interview	Lusaka Chongwe	89 14
Conditions tested	Tested when pregnant Partner was pregnant Daughter was pregnant Taken ill at health facility Circumcision Unspecified	55 13 5 22 3 5
Data collection method	In-depth Focus group	73 6 groups
Sex	Men Women	34 69
Total		103

^*^All participants in this study came from various mixed backgrounds. They were a mixture of mothers, housewives, churchgoers, Chongwe rural community members (villagers), women without children, married and unmarried men, researchers, police officers, a tax driver, lawyers, information technology (IT) specialists; workmen, subsistence farmers, a business consultant, church ministers, a nun, social workers, baby-sitters, sales assistants, a secretary, an ART adherence officer, a pharmacy technologist, etc.

#### Focus groups

We conducted six focus groups in total, which comprised the following groups of participants: churchgoers, midwives/nurses, women (including pregnant women), schoolteachers (men only), PLHIV, and Chongwe-rural (see table 1).

### Study Setting

The fieldwork was conducted in Lusaka and Chongwe. As the capital city, Lusaka is one of three provinces with the highest prevalence of HIV in Zambia and has a population more diverse than any other province. Chongwe is a largely rural community that provides a contrast to the diversity and urbanness of Lusaka.

Interviews were held in participants’ homes (the majority), health facilities, church premises, workplaces, and social establishments. The languages in which these were conducted were either English or Nyanja: the interviewer (the first author) is conversant in both languages.

### Sampling and Recruitment

All participants were purposively sampled to ensure information-rich data was obtained (Curtis et al. 2000; Patton 2002; Teddlie and Yu 2007; Palinkas, Horwitz, and Hoagwood 2015; Heckathorn and Cameron 2017). We adopted the purposive approach because this is often applied in studies where small samples are investigated, as the use of intense and focused approaches such as in-depth interviews offer a unique means of comprehending complex human behaviour (Gledhill, Abbey, and Schweitzer 2008). In line with this strategy, participants were identified, selected, and approached by the author on the basis of their sociocultural status and/or the fact that they were knowledgeable about or experienced with HIV testing and decision-making (Palinkas, Horwitz, and Hoagwood 2015). This explains the targeting of, for example, PLHIV, medical doctors, midwives and nurses, HIV counsellors and pregnant women who attend antenatal services, and spouses and caregivers. These participants were also in a position to communicate their experiences and views in an expressive, coherent, and reflective manner. These diverse but unique participants provided informed responses that could not have been acquired from other sources (Saunders 2012).

We also used snowballing sampling—a chain-referral method—to reach hard-to-access participants, such as PLHIV, medical doctors, and policymakers (Valerio, Rodriguez, and Turner 2016). For this strategy, the author asked friends, acquaintances, those already interviewed, potential participants, and family members to ask their acquaintances, friends, or family members (in short those from their social circles and community) if they would be willing to take part an interview. Individual interview appointments were then arranged with participants who expressed an interest and gave informed consent. All participants were aged eighteen and above.

### Interviewing Process

All interviews were conducted by the interviewer (the first author) in English (the majority) or Nyanja (a local language). Each lasted between thirty and 110 minutes. The interviews were either voice-recorded or recorded as field notes (Mack et al. 2005; Deggs and Hernandez 2018). The majority were audio-recorded and transcribed verbatim. Unrecorded interviews were captured using handwritten field notes (in circumstances where participants declined to be audio-recorded) and expanded within twenty-four hours. The interviewer took field notes during both recorded and unrecorded interviews.

All interviews were conducted with the help of adapted semi-structured interview guides containing a set of specific questions (see table 2). These were approved by an ethics committee in Zambia prior to use. During the interview, participants were asked questions relating to their experiences and understanding of informed consent and autonomy in HIV testing. To ensure that both the interviewer and participants were talking about the same thing, autonomy was defined for each participant as “self-governance.” Sample size was determined by data saturation. Field work took place during the first half of 2015. The project as a whole was completed in April 2018.

**Table 2:** Sample semi-structured questions later adapted and asked to different participants during interviews in Lusaka and Chongwe, Zambia

• *Have you ever tested or thought of testing for HIV?* • *What was/would be your motivation for testing for HIV/not testing? Why?* • *Before making the decision to test for HIV, did/would you discuss your wish to test with someone else prior to? Why/why not?* • *Do pregnant women have any moral responsibilities towards HIV testing? How/why?* • *What are your views on informed consent HIV testing policies/practices in Zambia? Why?* • *What would you say to a close relation/patient whom you may sincerely suspect may be HIV- positive due to their lifestyle/or are symptomatic and yet they refuse to test for HIV? How/why?* • *What does autonomy mean to you?* • *What does community mean to you?* • *What does knowing your/close relative HIV status mean to you?*	

### Ethical Considerations

The interviewer gave each participant an information sheet to read before the interview. This detailed the purpose, procedures, benefits, and risks of the study. For illiterate participants, the interviewer read all the relevant information contained in the information sheet and consent form by concurrently translating the information into Nyanja, a language all illiterate participants spoke and understood.

Birkbeck, University of London Ethics Board and ERES Converge IRB, Zambia, (a local Zambian ethics board) approved the research protocol and instruments, including the semi-structured interview questions. Written informed consent was obtained from all participants prior to the collection of data.

## Data Analysis

We conducted a hybrid analysis using both inductive and deductive approaches (Lacey and Luff 2007; Burnard et al. 2008; Swain 2018). Firstly, we conducted an inductive thematic analysis of data to develop codes. This involved repeatedly reading through transcripts to identify emerging and recurring themes. Secondly, the semi-structured interview guides were consulted to assess and confirm whether participants’ responses answered the research question(s). Thus, the coding process implemented in the generation of themes and subsequent consolidated findings involved systematically and manually re-reading transcripts and was informed by existing literature. This consequently led to the identification of themes, categories, patterns, and relationships from the data (O’Connor and Gibson 2003; Charmaz 2006; Thomas 2006; Burnard et al. 2008). The generated codes were verified and discussed between the authors and the first author’s supervisor to ensure consistency. Any discrepancies in codes were discussed until a consensus was reached.

The recurring themes that emerged from both interview and focus group discussion transcripts were taken as indicative of a shared understanding between participants (Flowers, Duncan, and Knussen 2003; Mack et al. 2005). The extracts presented in the results section were selected because they captured the recurrent themes expressed by the most articulate participants (Flowers, Duncan, and Knussen 2003). The finalization of the codes was an iterative process characterized by multiple rounds of data coding, reflection, consultation, and revision (Kasoka 2018).

The major themes that emerged were natural sociality, solidarity, cooperation and mutual responsibility and reciprocity, futurism and legacy, and religion. These are summarized as follows: 1) Natural sociality—human beings naturally born (without their will) into society and co-existing alongside other humans with whom they share common frailties and vulnerabilities (Gyekye 1997). 2) Solidarity—identification with others and working with and for each other to support the common good (Senghor 1964). 3) Cooperation, mutual responsibility, and reciprocity—these are interrelated terms which entail that a person does unto others as they would want others to do to them (mutual contribution) for both their own and the common good (Kaunda 1973; Tutu 1999; Masina 2000; Woods 2003; Gyekye 1997). 4) Futurism and legacy—respect, promotion, and protection of “a continuum of the dead, the living, and the yet unborn” to preserve future and collective good (Woods 2003). 5) Religion—belief in the presence of a high power that exercises rewards and punishment on the basis of, among other things, one’s faith and deeds towards God, oneself, and other human beings (Kaunda 1973; Tutu 1999; Gyekye 1997).

## Results

The five broad themes that emerged from the analysis suggest that participants view humans as inherently social beings, interrelated and interdependent—with a moral duty to promote the common good. Overall, participants recognized the rights of individuals but denied that a person should be described solely in terms of physical and psychological properties and have agency furnished upon them at the cost of the common good (Nussbaum 2003; Woods 2003). The results for each theme are presented in detail in the following sections.

### Natural Sociality

Participants described themselves as inescapably born into society and as social beings whose own well-being is inextricably linked to the well-being of others in their communities. They dismissed individualistic conceptions of autonomy. They expressed an ontology of natural human interconnectedness, interdependence, shared vulnerability, and common good, asserting that an individual cannot exist or survive outside the social setting in which he or she was born (Nussbaum 2003; Agulanna 2010).

The results in general indicate that participants were aware of different facets of human condition—being, identity, time, and space. They consequently advanced a perspective that favoured social living and responsibility over individualism, a legacy that can be safely attributed to dominant indigenous African civilization: a social order that colonialism, apartheid, and capitalism failed to supplant (An-Naim and Deng 1990; Masina 2000; Woods 2003; Maluleke 2012; Omaswa 2014).

Participants expressed how as individuals they are naturally born into and are one with society (Senghor 1964). They asserted that a human being can never be the unconditional master of themselves (autonomous) because they have neither complete control of themself nor of their environment (Kaunda 1973). Furthermore, they considered natural sociality and its consequences as a reality inescapable until death. They linked this with the need to promote communal good to ensure mutual well-being and survival:

… we can’t take ourselves out of the society. We are one and part of the society. Whatever happens in the society affects us in an indirect or direct way. Each and every other thing is affecting us because we are living in the society; there is no way that will take us out of the society until death. (HIV counsellor, male)

The community is part of us. … you cannot do anything without the community. The community affects our well-being. The community is a part of us. … Yes, we cannot do anything without the community. Because as human beings, we are interdependent. I depend on you; you depend on me. Have you seen this? For this interview, why didn’t you go to the animals? (Police officer, male)

Participants asserted that autonomy is achievable through other people. Thus, they suggested that a person is essentially a person through other people (Tutu 1999). They suggested that rational choice or self-reflection theories of autonomy, or any other self-determination theories that position a human as the sovereign of their choices and life, are incoherent at best (Kasoka 2018, 2020). Human life and well-being were viewed as naturally interlinked, including health and well-being (Jones 2020).

However, by suggesting that a person is “a person through other people,” they did not claim that individuals cannot make personal decisions. The participants did not disregard or ignore the status of the person as a unique and distinct human (Ravven 2013; Zahavi 2014). However, participants felt and thought that they can develop their potential and their originality and promote their well-being only in and through society, via a union with other people (Senghor 1964).

It’s true you can accept somebody living their life [as they wish]. But it depends again on the kind of decision that they are making. Sometimes, it’s important that you make them [individuals who exclusively embrace personal autonomy] understand that life is not about autonomy. Life is not about living my own life. Not at all … because no man is an island. You may say, “I am autonomous, I can live my own life,” but in this world, you do not live alone. You live amongst people; you live with people … People must begin to understand that life is not all only about me. (HIV Counsellor, male)

… we need to respect individuals’ rights. But, when other people’s lives are at risk, I think that’s a statement that probably needs to be rephrased. Children with HIV don’t deserve to experience what they are going through … especially if their mothers had the opportunity to actually prevent HIV. So, I think that it is a question of yes or no. We need to respect individual circumstances, individual rights, but those individual rights obviously go with responsibilities. For pregnant mothers, we probably need to rethink that issue for the sake of the unborn child and even for [the] sake of the mother who we are going to help by removing the burden of looking after a sick child …. (Medical doctor, female)

### Solidarity

Several participants claimed that the promotion of individuated autonomy is unknown in Zambia (Silavwe 1995) as traditional ethics and practice in the country are premised on solidarity (Kaunda 1973; Imasiku 2009; Kasoka 2018). Participants argued that informed consent requirements in HIV testing overlook the interlinked nature of personal and collective rights (Gaylin and Jennings 2003; Ravven 2013). They argued that because a person suffering from AIDS will inevitably depend on others for care by virtue of human interdependence, people should test for HIV in an effort to prevent any impact on the lives of others.

Participants stated that they look out for each other. The recognition of solidarity rights by a majority of the participants was motivated by personal concerns for the well-being of their family members, friends, and whole communities and an identification with shared humanity and its immanent frailty. They dismissed the ethics of universalized individual autonomy (Healy 2007) as incompatible with human natural sociality and harmful to their sense of community, unity, and common striving and thriving (Gyekye 1995, 1997):

We have attachments to patients. One will look at a patient and see a human being, so you want the best for that individual. Sometimes, you will have to put aside the policy [informed consent requirements in HIV testing] to help an individual. This is what happens in practice. In practice, we respond to a human face. We see individuals as having a family, and so they need to be helped. The person will even thank you later for doing the right thing for them. (HIV policymaker, male)

These people [HIV counsellors and midwives] who test us when we are pregnant help us. They are interested in our well-being. They are right to force us to test for HIV. They should be concerned about the well-being of my unborn child because when my child gets ill, the hospital will have to spend their time and resources to look after my child. To know one’s HIV status is to protect one’s child. Nurses say they feel bad when they see a mother with a child born HIV-positive. They say that they are also mothers, so they feel the pain the mother of an HIV-positive child may feel. (Mother with three children, female)

We are Africans…. You just say “*Ubuntu*!” Some of us just love people. And even in some days, I am always the last to leave here [the office] because I have so much work to do…. Somebody even said, “since you have got everything, why do you work so late?” …. People think that if you are rich, you have a house, you have a car, then that is enough. But other people think that I am making a difference in someone’s life. (Medical doctor, female)

This [individual autonomy without solidarity] is totally a question of a borrowed bucket. We are drawing water using a borrowed bucket. As you know, when we have borrowed a bucket, we are not going to put in enough water for fear; sometimes, we may not know how to carry the bucket properly. The type of consent we have [in HIV testing], informed consent, is borrowed. Of course, it is founded on the principles of universal human rights. But we have a culture; we have a way of living. I just gave you an example of the rural setting; people don’t exist as individual beings. They exist as families…. And we shouldn’t be judged as primitive or otherwise. That’s just the way we are. And that way should be respected. (Medical doctor, male)

### Cooperation, Mutual Responsibility, and Reciprocity

Participants argued that the current regime of human rights and medical ethics is biased towards individual self-centredness. Individualism was viewed as “diminish[ing] the importance of caring, reciprocity, community building, generosity and cooperation” (Healy 2007, 24). They suggested that human rights and ethics that are exclusively based on individual autonomy tend to overlook the fact that “no society can rest on autonomy alone”: participants stated that “our humanity is fulfilled by conjoining rights and responsibilities, autonomy and relationships, independence and interdependence” (Gaylin and Jennings 2003, 67).

Participants vouched that a life lived as a member of a community promotes an altruistic ethos of cooperation, mutual responsibility, and reciprocity. In this regard, they viewed individual humans as social beings who need society and all that is offered within its social structure in order to grow, aspire, and achieve a worthwhile life. As David Kenneth Kaunda, Zambia’s first president, once noted: “we can neither applaud nor tolerate irresponsible and wholly selfish individual development”; “freedom without moral commitment is aimless and self-destructive” (Kaunda 1973, 29).

Participants viewed cooperation, mutual responsibility, reciprocity, compassion, concern for others, and harmony as key to human dignity, not individual autonomy (Nussbaum 2003; Gyekye 1997):

Living with your neighbour is a good thing. This is because if I am sick, my neighbour will care for me. If I am suffering, my neighbour will help me; if I have no salt, my neighbour will give me salt. If there is anything we are lacking, we will help each other. So, it is a good thing to live amongst people, to live with people, to cooperate with people. If you live alone, you will be sick and you will die on your own. You will not have anyone to care for you. (Chongwe, focus group, mixed-male and female group)

I learn a lot of things from the community. The community teaches me. A community that works together and is cooperated and united … teaches me to be organized, to be united with other people…. Now, I am a counsellor; I also contribute to the community because it is very important for me…. For the community to develop everybody, they have to contribute to the development of the community. So, my contribution to the community as a counsellor is my information. The information that I have now, I share it with the community. My knowledge about [HIV] testing and counselling, I share it with the community. I don’t only build myself; I also build a community…. (HIV counsellor, male)

### Futurism and Legacy

In contrast to the ethics of universalized individual autonomy which protects and promotes an individual’s right to fulfil their desires and well-being with virtually no regard for a common legacy, we found that the majority of participants expressed views that were not exclusively grounded in the immediate satisfaction of an individual’s autonomy. In addition to securing their present well-being and rights, a large majority of participants were more concerned with the preservation of future well-being and future generations and leaving a legacy that promotes and protects the common good (Senghor 1964; Kaunda 1973; Kenyatta 2015; Woods 2003). Thus, their concept of autonomy, human rights, and well-being was also future-focused.

The following quotations highlight views expressed by participants that the protection of future enjoyment of rights and well-being (Woods 2003) is important to them. They believe that individuals and communities of people have a moral duty to prevent future suffering and deaths, therefore HIV testing is perceived as a common good:

- *Participant 1*: Their rights [individual autonomy] could be there which is quite alright. But, if you know that if you don’t test a given person they are going to die, it is better we put aside the rights.
- *Participant 2*: Because if you are going to die, that right [autonomy] won’t be there. (PLHIV, focus group, mixed-male and female group)

Regarding HIV testing … I want people to be healthy in the community. Testing for HIV in the family/community will lessen unnecessary deaths. In this manner, my community will not go into extinction because of AIDS. Individuals, even when they can claim individual rights to make choices, do not live in a vacuum. Their daily decisions have an impact on the community in which they live. When I say community, this also includes the country. Zambia, as a nation, is a community of people. (Police officer, male)

Regarding the testing of pregnant women, the majority of participants argued that this is acceptable because compulsorily testing such women for HIV would eventually lead to both the mother and child living a healthy life, which is good for the preservation of future generations. For instance, a housewife said that if her own daughter, who is above the age of consent, refused to test for HIV, she would secretly ask her work colleague to test her. Accordingly, with respect to human rights, the interviewer asked her “what then happens to respecting her daughter’s autonomy,” to which she responded:

… because I want to protect the unborn child. Also, all the problems that my daughter will face will eventually be my problems. If my daughter refuses to listen to what I tell her, then I will tell her to get out of my sight. My daughter has rights, but she will forget about them when she is in trouble. She will come rushing to me seeking help… (Mother/housewife, female)

Responding to a similar hypothetical scenario where a pregnant daughter refuses to test for HIV and invokes her right to autonomy, one participant in the women’s focus group answered:

On that, I will emphasize that “yes, they are your rights. But, if you get sick, it is me who is going to suffer as a mother”…. “I am a widow; I am not working. Where can I find 13 million to buy your CD4 count [sic] at UTH? This will mean that you will die. And if you die, the funeral expenses will be 10,000 Zambian Kwacha.” So, I will tell her that “yes, they are your rights, but you need to consider the implications of your decision on yourself, your child, and me as your parent ….” (Women, focus group, women-only group)

Studies and media reports (Mutombo 2007; Southern African News 2014; Phiri 2015; Kasoka 2018) have claimed that people, particularly pregnant women, are tested without informed consent. One of the reasons invoked for this is that healthcare providers do so to protect the lives of unborn children. This suggests that pregnant women’s autonomy is not prioritized, even in the light of consent requirements as per Zambia’s HIV law and policies.

### Religion

The lived experiences and perspectives of participants in Lusaka and Chongwe are influenced not only by sub-Saharan African communitarian and moral thought but also by Christian ontology (Carmody 2007). Several participants referenced their Christian faith during interviews. This is perhaps unsurprising. A large number of African people are deeply religious. Even before the colonization and subsequent Christianization of Zambia, religion was central to African life, culture, and community (Kaunda 1973; Gyekye 1995; Colson 2006). Several responses by participants suggested that Christianity mandates and motivates them to respond to the prevalence of HIV in a manner that preserves human life. They indicated that because Christians are the collective body of Jesus Christ, they have a God given obligation to act in the interest of each other for the glory of God:

It [caring for others and being responsible by testing for HIV] actually is a Christian mandate … as God’s people, it’s a God-given responsibility to take care of life, and by that, we look not only at the spiritual part but also the physical [well-being]. … Christ, our Lord, gave the best example. He was concerned about the spiritual as well as the physical. The physical as demonstrated in the way that he feeds the hungry, heals the sick …. (Senior economist/church elder, male)

80 per cent of the people that live in Zambia are Christians. We feel for each other, and like I said earlier, if you are not considering the importance of helping each other, we cannot develop as a country. We will never develop as a country…. (Teachers, focus group, male-only group)

## Discussion

This study explored the importance of personal autonomy in decision-making amongst people living in Lusaka and Chongwe, specifically within the context of universalized informed consent requirements in HIV testing. The study was designed to determine how personal autonomy is viewed in a local context, in this case Zambia.

The results indicate that nearly all participants viewed themselves, in the context of their lived experiences and perspectives, as born into the society; they perceived themselves as socialized, interconnected, and interdependent beings who have ontological reasons and a moral duty to promote the common good in order to enable mutual well-being and survival. The results also demonstrate that participants regarded human well-being as not merely individual but also social (Jones 2020) and thus considered HIV testing premised exclusively on personal autonomy as inappropriate/unjustifiable in their communities.

They reject ethical assumptions that “do not acknowledge the deeply relational and embedded reality of the human condition which inevitably shapes decision making”, and therefore constitute autonomy in real life (Milligan and Jones 2016, 21). Hardly any participants viewed themselves as being solely responsible for defining “one’s own concept of existence, meaning, the universe, and the mystery of human life” (*Planned Parenthood of SouthEastern PA. v. Casey* (1992), ¶20).

Their views on the nature of personhood in HIV testing are consistent with the dominant traditional African view which denies that any single human being can be described solely in terms of physical and psychological properties (Senghor 1964; Kaunda 1973; Kwesi 1977; Donnelly 1990; Gyekye 1995, 1997; Tutu 1999; Taylor 2018; Agulanna 2010; Denison et al. 2014; Malunga 2014; Kenyatta 2015). It is important to note that we have used the adjective “dominant” when referring to sub-Saharan African ontology to avoid simplifying the way of life of sub-Saharan African peoples as having a communitarian outlook. Our understanding in the light of available literature is that communitarian ethics are a dominant, persisting, and shared ethical position for the majority of people in sub-Saharan Africa (Gyekye 1995, 1997; Agulanna 2010). We are aware that not every person living in sub-Saharan Africa necessarily holds a homologous ontological outlook (Oyugi and Gitonga 1987).

Although participants generally suggested that the exercise of personal autonomy in HIV testing is acceptable in certain circumstances, the dominant view was that individuals should not strictly adhere to such a value in all circumstances. For example, they argued that doing so would be especially detrimental to fetal “rights,” injure the welfare of one’s immediate family, impede correct diagnosis and treatment, hinder appropriate national health planning, annihilate whole communities, and harm the economic and sociocultural well-being of their communities and country.

Participants suggested that by virtue of natural human interdependence, sociality, and interconnectedness, there are limits to how far a person’s desires, decisions, or actions should be upheld as fundamental. According to this view, “human beings have dignity by virtue of their capacity for community, understood as the combination of identifying with others and exhibiting solidarity with them” (Metz 2011, 532). This ontological and moral view of personhood repudiates any description of a person solely in terms of their physical and psychological properties (Nussbaum 2003).

We agree with the participants that personal autonomy ethics inspire individual solitariness, self-centredness, and self-actualization, often at the cost of the pursuit of the common good. Promoting the primacy of personal autonomy detaches an individual from his or her natural sociality, interconnectedness, and interdependence with other people. Such an approach neglects to acknowledge and protect the embedded, interlinked, and corporate nature of human existence.

The evolution of self-determination from city autonomy to individual rights (Schuck 1994; O’Neill 2003; Jackson 2013) has broadened the rights of individual persons almost to the extent of totally denigrating individuals’ moral obligation to the common good (Gaylin and Jennings 2003). Indeed, even “[t]he great philosopher of medicine, Hippocrates, would be shocked by the world of modern medicine and possibly hounded out of the medical profession and driven into poverty by” the current universalized consent requirements in HIV testing that are premised on individualism (Kurtz 2000, 1243).

The individual, who is embedded in a complex social and cultural environment and whose illness (if they are living with HIV) could affect other people, is framed as the sole author of his or her own medical treatment and life choices. They are told that they can choose to test or not to test because they are their own person and the author of their own life and destiny. Individuals’ rights and course of actions are primary, while collective rights are only recognized to the extent they support the enjoyment and protection of an individual’s rights and choices (Woods 2003).

Such an outlook represents an understanding to the effect that to be a human being is to demand protection and require help from others. Ironically, social responsibility (even when some international human rights instruments imply that human beings are interdependent) is relegated to mere interference with the value of an individual’s autonomy. Other human beings are expected to mind their own business because only the person whose autonomy we are enjoined to respect and celebrate has the right to “define the nature of her relationships with others” (Schuck 1994, 900).

In other words, an individual is epitomized and celebrated as a moral agent whose rational and moral choices need to be recognized and respected. Consequently, individual rights become individual claims to be protected from the interventions of other people (Niekerk 1998), who are inescapably directly or indirectly affected by their personal choices. It is troubling to appreciate that a person who is naturally dependent, vulnerable, fallible, and a moral agent (Ravven 2013; Kasoka 2020) is now proclaimed and celebrated as a rational and/or sovereign agent and their own person; it implies they are empowered by their inherent autonomy to choose and direct their own HIV medical therapy without interference from others.

A traditional African communitarian adherent would believe that “an injury to one person is an injury to all,” yet at the same time hold that the survival and well-being of the community is dependent upon the well-being of the individual (Elechi, Morris, and Schauer 2010, 75). Put differently, the traditional sub-Saharan African concept of personhood and human rights recognizes and promotes both the humanity of each person and the entitlement of other “people to unconditional respect, value and acceptance from one’s community” (Himonga, Taylor, and Pope 2013, 379). It holds that “every person has a corresponding duty to show the same respect, dignity, value and acceptance to each member of that community” (Himonga, Taylor, and Pope 2013, 379–380). This outlook on personhood advances a concept of co-responsibility and mutual enjoyment of human rights, as well as a human whose capacity for autonomy is only achievable through other people and God (or gods).

When we argue for a conception of social autonomy, we do not imply that individuals who refuse to test for HIV should be ruled by the appetites of the majority to ensure they test no matter what. Instead, the implication of our analysis is that socio-relational conceptions of autonomy are more philosophically and morally convincing than individualistic ones (Kasoka 2020). We appreciate that human beings are unique and distinct—physically, emotionally, mentally, and morally (Ravven 2013). However, we also recognize that humans are only able to exercise such uniqueness or their distinct self in a social context by virtue of human natural sociality, interdependence, and interconnectedness:

… the self is where our identity resides. It is the medium through which our actions are guided and our world is perceived … This is clear even from the dictionary definitions of self: “the total, essential, or particular being of a person,” or “the essential qualities distinguishing one person from another,” or “one’s consciousness of one’s own being or identity; the ego.”… [However] The self is not a truly autonomous ego, it is an interactive entity defined not just in terms of differences from others but in relationship to them. (Gaylin and Jennings 2003,145–146)

Thus, with regard to HIV testing policies in Zambia and other sub-Saharan African countries, HIV policymakers should arguably take heed of Darley, Luethge, and Blankson’s (2013) conclusion that, due to the sub-Saharan African communal culture of belongingness, advertising appeals in sub-Saharan African countries should be focused on presenting the individual as a member of the community.

The doctrine of personhood invoked by participants in the present study has the realistic potential to provide an appropriate ethical premise and “foundation for a human rights paradigm that is capable of addressing the broad panoply of claims that are vital to the full attainment of human dignity” (Woods 2003, 55–56).

We suggest that rather than basing informed consent requirements on personal autonomy, it is shared human vulnerability, common human frailty, interdependence, love, friendship, and trust that should lie at the core of ethical decision-making in HIV testing in Zambia, and indeed across sub-Saharan Africa (Kasoka 2020). This serves to reframe autonomy in HIV testing in a way that deliberately acknowledges the illusion of personal autonomy and “considers [that] the unique moral frameworks, relationships, and cultures of individuals can provide a more ethically sensitive,” respectful, and realistic basis for decision making in HIV testing (Milligan and Jones 2016, 21).

## Limitations

The findings in the current study do not necessarily represent the views of all people in Zambia as fieldwork was limited to Lusaka and Chongwe. Studies conducted in other parts of the country may therefore yield different results. The aim of this study was essentially to gain in-depth, rich, and localized empirical data on what autonomy in HIV testing means to different people (Polit and Beck 2010).

## Conclusion

Participants from Lusaka and Chongwe appear to have sourced their views of autonomy from a dominant traditional sub-Saharan African ontological and moral philosophy which is not—in strict terms—reflected in HIV testing policies in Zambia. They viewed interdependence, not autonomy, as an essential characteristic of the human condition (Woods 2003).
